# Description of a new species of coral-inhabiting barnacle, *Darwiniella angularis* sp. n. (Cirripedia, Pyrgomatidae) from Taiwan

**DOI:** 10.3897/zookeys.214.3291

**Published:** 2012-08-07

**Authors:** Yi-Yang Chen, Hsiu-Chin Lin, Benny K.K. Chan

**Affiliations:** 1 Institute of Ecology and Evolutionary Biology, National Taiwan University, Taipei 106, Taiwan; 2 Biodiversity Research Center, Academia Sinica, Taipei 115, Taiwan

**Keywords:** Barnacles, Corals, Pyrgomatidae, cryptic diversity, Symbiosis, host-specificity

## Abstract

The present study has identified a new species from the previously monotypic genus *Darwiniella* Anderson, 1992. *Darwiniella angularis* **sp. n.** is similar to *Darwiniella conjugatum* (Darwin, 1854) in external shell morphology and arthropodal characters. *Darwiniella conjugatum*, however, has a sharper tergal spur and a less obvious adductor plate angle when compared to *Darwiniella angularis* **sp. n.** Molecular analyses on mitochondrial DNA 12S rDNA and COI regions also support the morphological differences. Sequence divergences in 12S rDNA and COI between *Darwiniella conjugatum* and *Darwiniella angularis* **sp. n.** are 5% and 13% respectively, which are equivalent to the inter-specific sequence divergences in other barnacles. Both *Darwiniella* species are common on *Cyphastrea* Milne-Edwards and Haime, 1848 corals and *Darwiniella angularis* **sp. n.** is also collected from *Astreopora* de Blainville, 1830 corals in Taiwan.

## Introduction

The coral-inhabiting barnacles of the genus *Darwiniella* Anderson, 1992 is a member of the family Pyrgomatidae, which has a symbiotic relationship with host corals. *Darwiniella* was considered to be a monotypic genus, represented by *Darwiniella conjugatum* (Darwin, 1854).

*Darwiniella conjugatum* was originally described by Darwin (1854) as *Pyrgoma conjugatum*. The species has a fused shell wall and a pair of fused opercular plates (scutum and tergum). Ross and Newman (1973) erected the genus *Nobia* to accommodate coral-inhabiting barnacles with fused shell plates and fused opercular valves, thus transferred *Pyrgoma conjugatum* to *Nobia conjugatum*. However, *Nobia conjugatum* has a deep adductor plate and a distinct tergal spur, which is different from all *Nobia* species. Subsequently, Anderson (1992) erected the new genus *Darwiniella* to accommodate *Nobia conjugatum* and defined the genus as “ Wall flat, fused; sheath occupying about half inner wall; scutum and tergum calcified together, without visible line of juncture; deep adductor plate and conspicuous rostral tooth; elongate spur; basis oval, deep.” However, Anderson (1992) had not formally assigned the type species of *Darwiniella* and Ross (1999) designated the type species of *Darwiniella* as *Darwiniella conjugatum* (Darwin, 1854).

In the present study, we collected a large number of *Darwiniella* specimens from coral reefs in Taiwan. Based on molecular analysis of the mitochondrial 12S rDNA (12S) and DNA barcode gene fragment (COI), we revealed two species of *Darwiniella*, *Darwiniella conjugatum* and an undescribed species. We used scanning electron microscopy (SEM) to examine the shell parts and light microscopy (LM) to examine the arthropodal characters of *Darwiniella conjugatum* and the *Darwiniella* sp. n. and both species were described herein from Taiwan.

## Materials and methods

### Specimen sampling and morphological analysis

**Figure 1.**
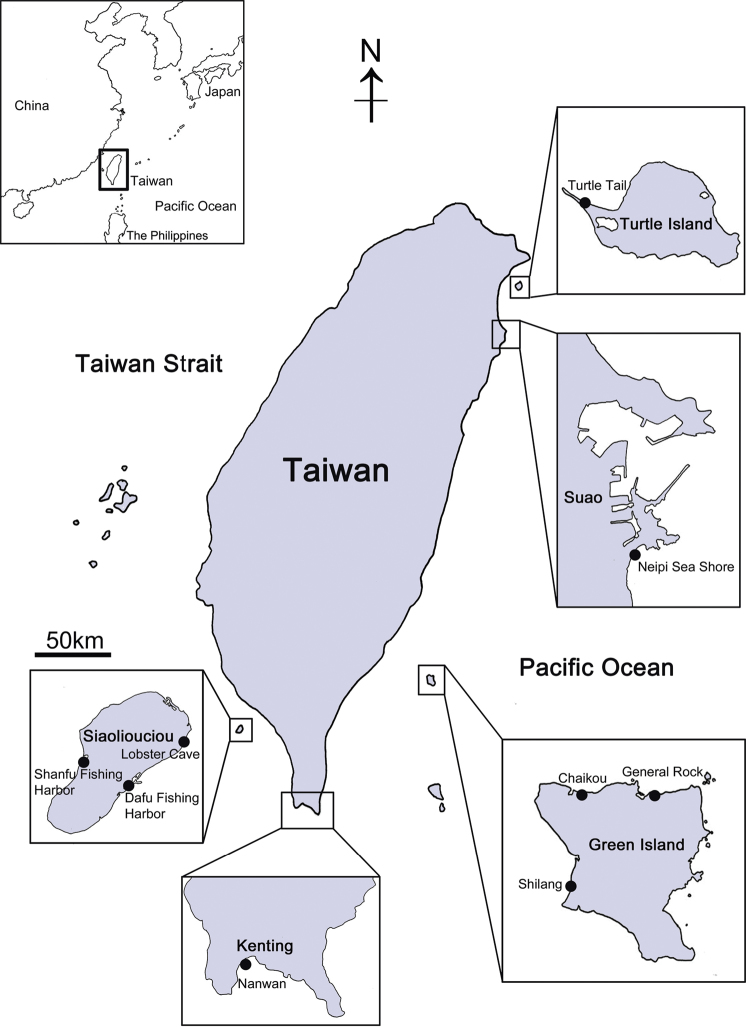
Collection sites of coral-inhabiting barnacle *Darwiniella* in Taiwan.

Sampling of *Darwiniella* specimens was conducted at the main island of Taiwan (Suao and Kenting) and outlying islands (Turtle Island, Green Island and Siaoliouciou Island; see Fig. 1 and on-line Appendix 1: Table 1 for site and location details). Small pieces of coral with barnacles embedded were collected with hammers and chisels at 5–20 m depth by SCUBA diving. All barnacles and host corals were preserved in 95% EtOH. Type and paratype specimens are stored in the National Museum of Natural Science, Taichung, Taiwan (NMNS) and the Biodiversity Museum of the Academia Sinica, Taipei, Taiwan (ASIZCR), and additional specimens in the barnacle collections in the Coastal Ecology Laboratory, Academia Sinica, Taiwan (CEL). Barnacles were isolated from the host corals using forceps and the morphological characters of shell parts (shells, scutum and tergum) and somatic bodies (6 pairs of cirri, penis and oral cone) were examined. Organic debris and coral tissue on the surface of shells, scutum and tergum were removed with forceps and further cleaned ultrasonically (2–5 seconds). Cleaned shells and opercular valves (scutum and tergum) were immersed in 1.5% bleach for about five hours to completely digest organic tissue and the shells were rinsed by slow running purified water (30 minutes) and air-dried. The shells, scutum and tergum were gold coated and observed under SEM, following the methods of Achituv et al. (2009). Cirri, penis and oral cone were dissected from the somatic bodies and examined under LM (Zeiss Scope A1) with high definition lenses (Zeiss Plan APOChromat 40X/0.95 and ZEISS Plan APO Chromat 100x/1.4 oil), which allowed clear observation of setal types on cirri and mouth parts. Setal descriptions follow Chan et al. (2008).

## Molecular analysis

Total genomic DNA was extracted from soft tissue of 110 *Darwiniella* specimens using Qiagen (Chatsworth, CA) QIAquick Tissue Kit following the manufacturer’s instructions. Partial sequences of mitochondrial genes 12S rDNA (12S) and cytochrome c oxidase subunit I (COI) were amplified by polymerase chain reaction (PCR) with primers 12S-FB and 12S-R2 (Tsang et al. 2009), and COI-F5 5’ AAACCTATAGCCTTCAAAGCT 3’ and COI-R4 5’ GTATCHACRTCYATWCCTACHG 3’, respectively. The PCR solution contained 40 ng of template DNA, 5 μl Taq DNA Polymerase Master Mix (1.5 mM MgCl_2_; Ampliqon, Denmark), 1 μM of each primer, and ddH_2_O with a final volume of 10 μl. The PCR reaction was conducted under the following conditions: 2 min at 95°C for initial denaturing, 35 cycles of 30 sec at 95°C, 1 min at 48°C, 1 min at 72°C with a final extension for 5 min at 72°C. The PCR products were then purified using the DNA Gel purification kit (Tri-I Biotech, Taipei, Taiwan). Direct sequencing of the purified PCR products was performed on an ABI 3730XL Genetic Analyzer with BigDye terminator cycle sequencing reagents (Applied Biosystems, Foster City, California, USA). Sequences were then aligned with CLUSTAL X (Thompson et al. 1997) using default settings and adjusted by eye in MacClade 4.07 (Maddison and Maddison 2005).

The genealogical relationships of specimens based on 12S and COI were inferred using K2P model and 1000-replicate Neighbor-Joining (NJ) method implemented in MEGA v5.05 (Tamura et al. 2011). Two specimens of coral barnacle *Hiroa stubbingsi* Ross & Newman, 1973 (GenBank accession numbers 12S: JQ946198, JQ946212; COI: JQ946237, JQ946244) were applied as the outgroup. The evolutionary distance (number of base differences per site) between sequence pairs were calculated with uncorrected p-distance and Kimura-2-parameter (K2P) models by MEGA.

## Results

### Systematics. Suborder Balanomorpha Pilsbry, 1916. Family Pyrgomatidae Gray, 1825. Subfamily Pyrgomatinae Gray, 1825. Genus *Darwiniella* Anderson, 1992

#### Darwiniella angularis sp. n.

urn:lsid:zoobank.org:act:FF17801C-71BB-4B1C-BF4B-F20AB02733B7

http://species-id.net/wiki/Darwiniella_angularis

**Figure 2.**
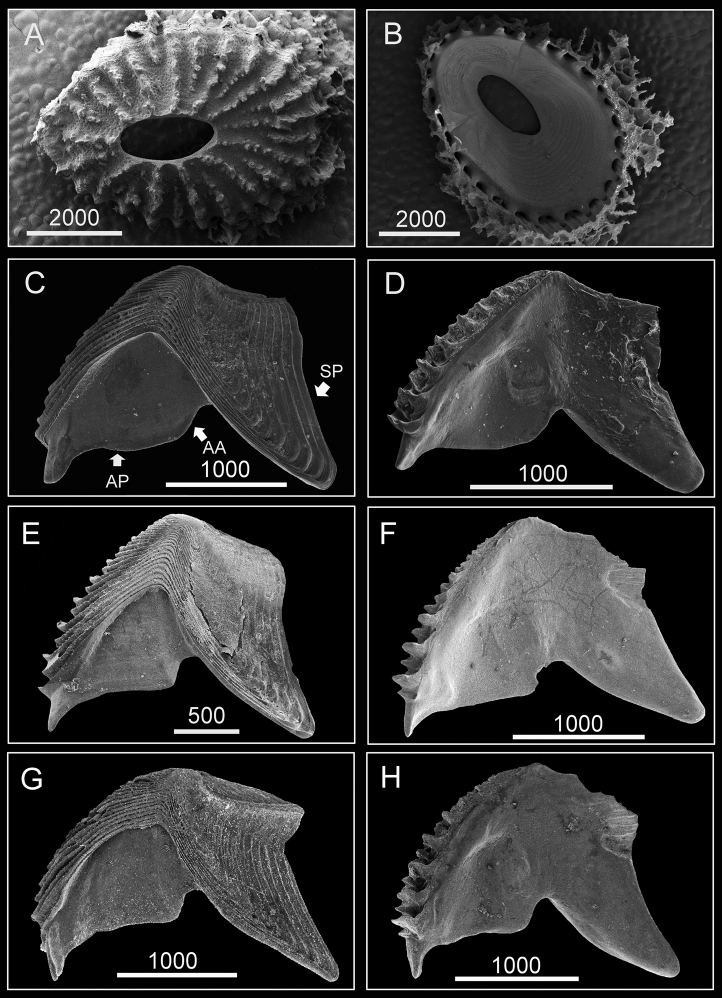
Hard parts of *Darwiniella angularis* sp. n. **A** Dorsal view of shell (NMNS-6878-001) **B** Ventral view of shell (NMNS-6878-001) **C** Dorsal view of fused scutum and tergum (NMNS-6878-001) **D** Ventral view of fused scutum and tergum (NMNS-6878-001) **E** Dorsal view of fused scutum and tergum (ASIZCR000203) **F** Ventral view of fused scutum and tergum (ASIZCR000203) **G** Dorsal view of fused scutum and tergum (additional individual, CEL-RYU-13-3) **H** Ventral view of fused scutum and tergum (additional individual, CEL-RYU-13-3). (AP: adductor plate, AA: adductor plate angle, SP: spur, scale bar: μm). Note the AA and SP of *Darwiniella angularis* sp. n. distinguish it from *Darwiniella conjugatum* (Darwin, 1854) (see Figure 10).

Figures 2 [Fig F3] [Fig F4] [Fig F5] [Fig F6] [Fig F7] [Fig F8] –9

##### Material examined.

HOLOTYPE. NMNS-6878-001, Lobster Cave, Siaoliouciou Island, Taiwan (22°20'N, 120°23'E), August 2010, coll. B.K.K. Chan, on host coral *Astreopora* sp. PARATYPES. ASIZCR000202, Turtle Tail, Turtle Island, Taiwan (24°50'N, 121°56'E), October 2010, coll. B.K.K. Chan, on coral host *Cyphastrea chalcidicum* (Forskål, 1775). ASIZCR000204, data same as ASIZCR000202. CEL-RYU-13-2 and CEL-RYU-13-3 data same as holotype. CEL-TI-1-7, CEL-TI-5-6, and CEL-TI-9-10 data same as paratype ASIZCR000202.

##### Diagnosis.

Scutum triangular, rostral tooth and obvious adductor plate present, basal margin of adductor plate forming obvious angle. Tergum trapezoid, spur triangular, medial furrow curved.

##### Description.

(Type specimen: 6 mm in basal diameter, rostro-carinal diameter to 5.2 mm). Shell plates fully fused, purple, oval, externally surface with about 24 strip-like projections radiating from subcentral orifice to shell plate margin (Fig. 2A). Bases of shell with about 27 internal ribs radiating from rim of inner operculum (Fig. 2B). Orifice oval, long, narrow, about 1/3 length of rostro-carinal diameter.

Scutum and tergum white, fused without junctions (consistent through 3 specimens, Fig. 2C–H). Scutum triangular, width equal to height, occludent margin slightly curved, rostral tooth basally, 15 teeth along ventral surface of occludent margin, tooth size increasing gradually from apex to base. Ventral view with oval-shaped adductor muscle scar (Fig. 2D, F, H). Dorsal view with obvious adductor plate, convex, extending below basal margin of scutum, plate about 3/5 height of scutum, basal margin with obvious angle (Fig. 2C, E, G). Dorsal surface with horizontal striations, striations with row of small pores.

Tergum trapezoid, lateral depressor muscle crests present. Spur triangular, blunt, curved, height reaching more than 1/2 height of tergum, basal margin not obvious due to curved spur. Dorsal surface with medial furrow, curving from basal margin towards the carinal margin (Fig. 2C, E, G). Dorsal surface with horizontal striations, striations with row of small pores.

**Figure 3. F3:**
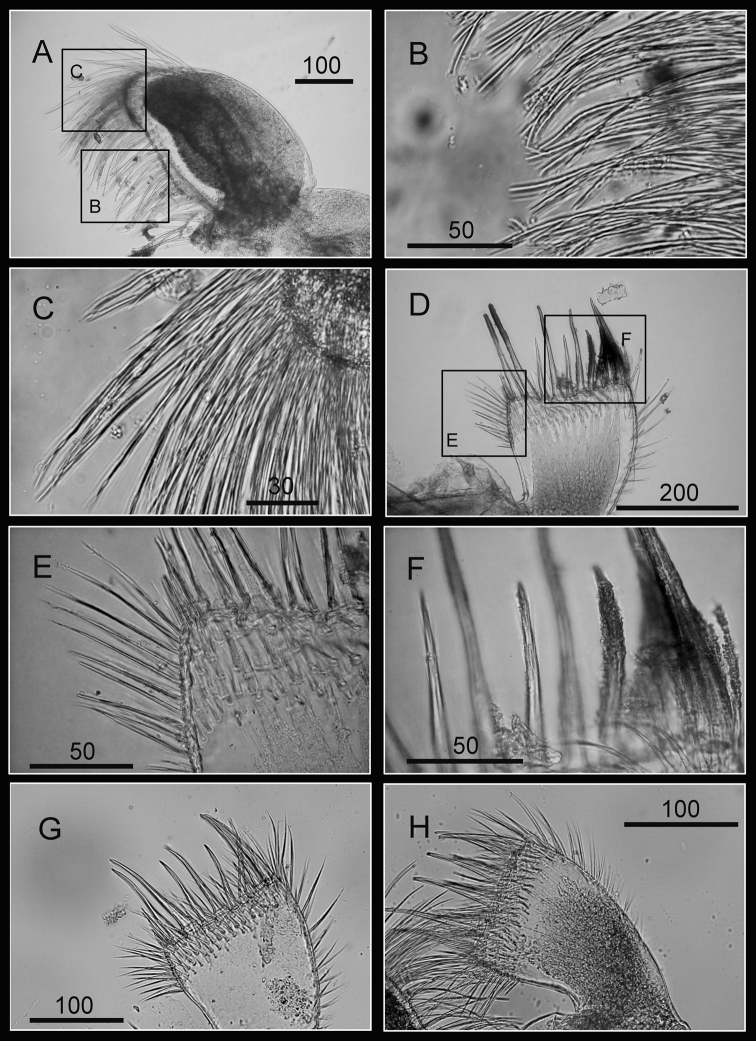
Oral cone of *Darwiniella angularis* sp. n. **A** Maxilla (NMNS-6878-001) **B** Serrulate setae on apex **C** Serrulate setae on inferior margin **D** Maxillule (NMNS-6878-001) **E** Simple setae on posterior margin **F** Large simple setae on cutting edge **G** Maxillule (additional specimen, ASIZCR000202) **H** Maxillule (additional specimen, CEL-TI-9-10). (scale bar: μm)

**Figure 4. F4:**
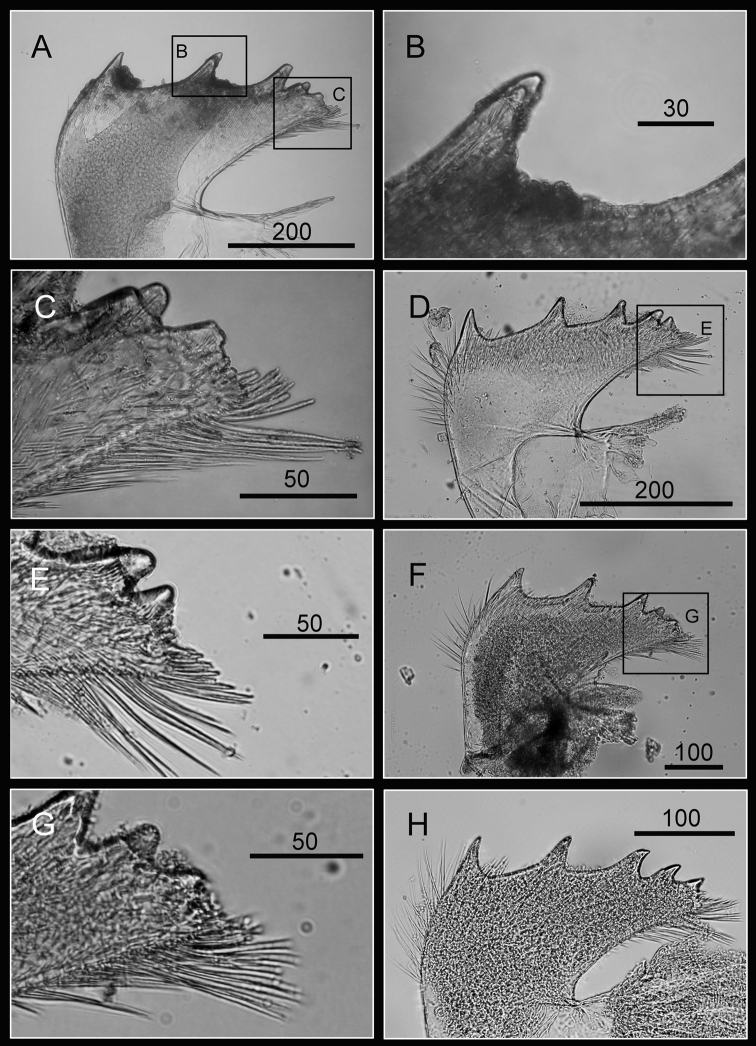
Oral cone of *Darwiniella angularis* sp. n. **A** Mandible (NMNS-6878-001) **B** Bidentate second tooth **C** Inferior angle with simple seta **D** Mandible (additional specimen, ASIZCR000202) **E** Inferior angle with simple seta **F** Mandible (additional specimen, CEL-TI-9-10) **G** Inferior angle with simple seta **H** Mandible (additional specimen, ASIZCR000204). (scale bar: μm)

**Figure 5. F5:**
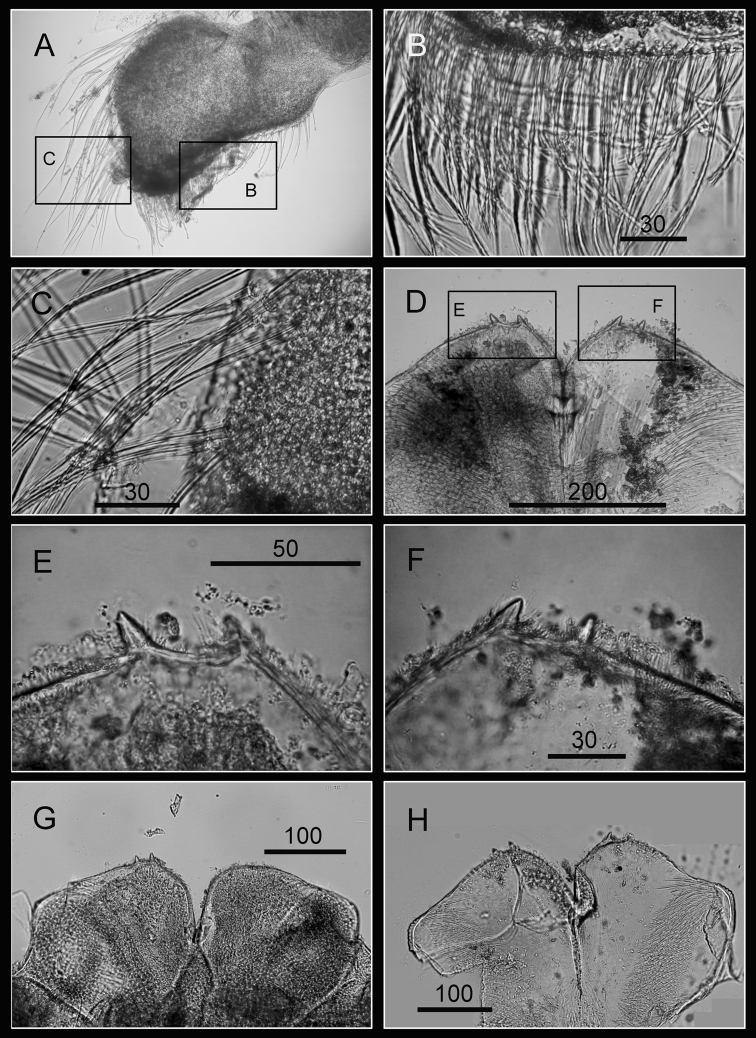
Oral cone of *Darwiniella angularis* sp. n. **A** Mandibular palp (NMNS-6878-001) **B** Serrulate setae on inferior margin **C** Serrulate setae distally **D** Labrum (NMNS-6878-001) **E** Teeth on labrum **F** Teeth on labrum **G** Labrum (additional specimen, CEL-TI-9-10) **H** Labrum (additional specimen, ASIZCR000202). (scale bar: μm)

Maxilla bilobed (Fig. 3A), serrulate setae distally (Fig. 3C) and along inferior margin (Fig. 3B). Maxillule cutting edge straight, without notch, bearing row of 7 large and 3 smaller setae (Fig. 3D). Region close to cutting edge with dense fine simple setae (Fig. 3F), anterior and posterior margins with long simple setae (Fig. 3E). Mandible with 4 or 5 teeth, excluding inferior angle (inconsistent in 4 specimens, Fig. 4A, D, F, H). Second, third and fourth teeth bidentate (Fig. 4B), the first 3 teeth occupying most of length of cutting edge. Lateral surface, lower margin and cutting edge of mandible bearing simple setae. Lower margin short, about 1/16 length of mandible, inferior angle ending in blunt angle with dense, fine setae (Fig. 4C, E, G). Mandibular palp rectangular, elongated (Fig. 5A), bearing serrulate setae distally (Fig. 5C) and inferior margin (Fig. 5B). Labrum bilobed, V-shaped notch between lobes, no or 2 sharp teeth on each side of notch (inconsistent in three specimens, Fig. 5D, G, H).

**Figure 6. F6:**
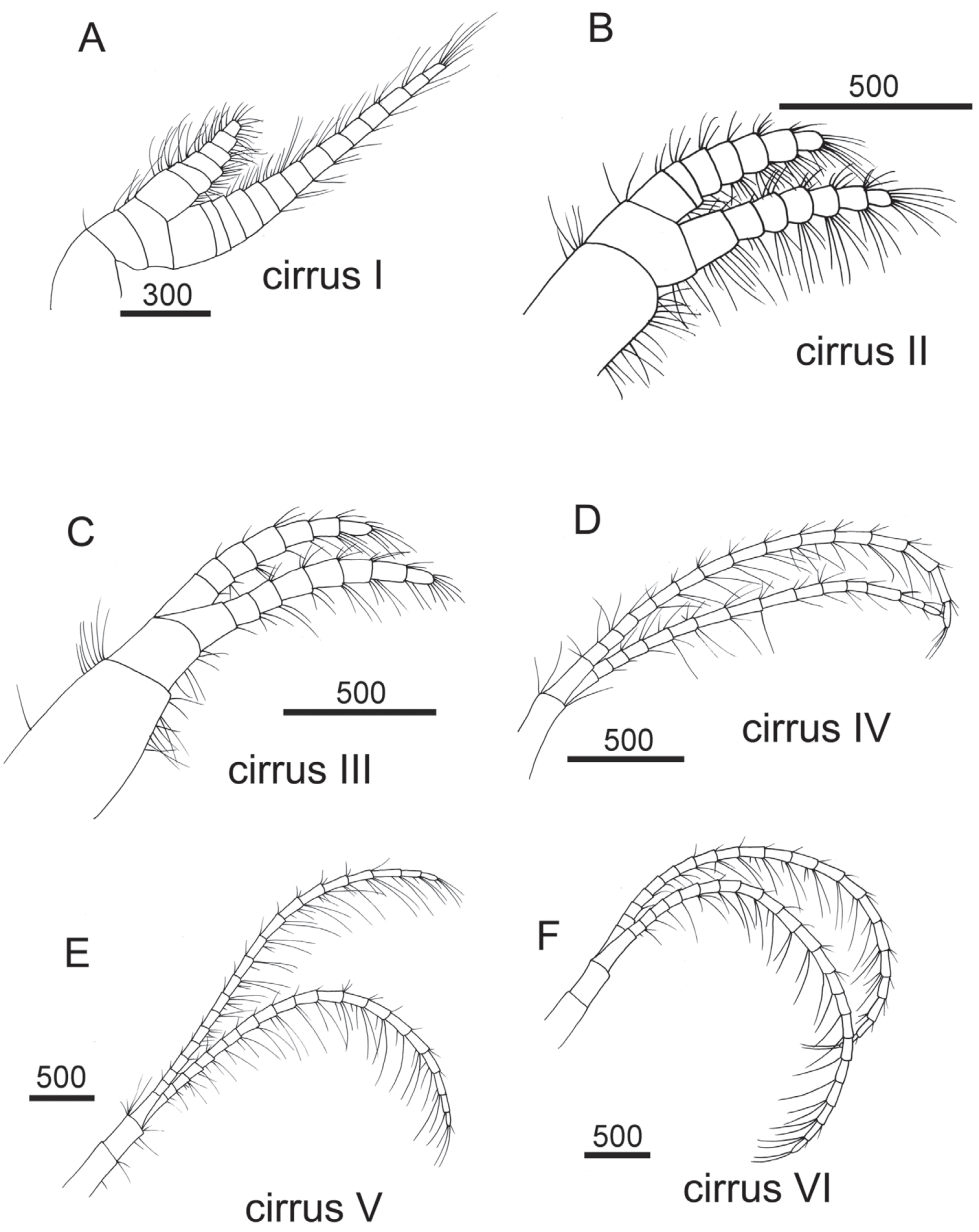
Line drawing of *Darwiniella angularis* sp. n. **A** Cirrus I **B** Cirrus II **C** Cirrus III **D** Cirrus IV **E** Cirrus V **F** Cirrus VI. (scale bar: μm)

**Figure 7. F7:**
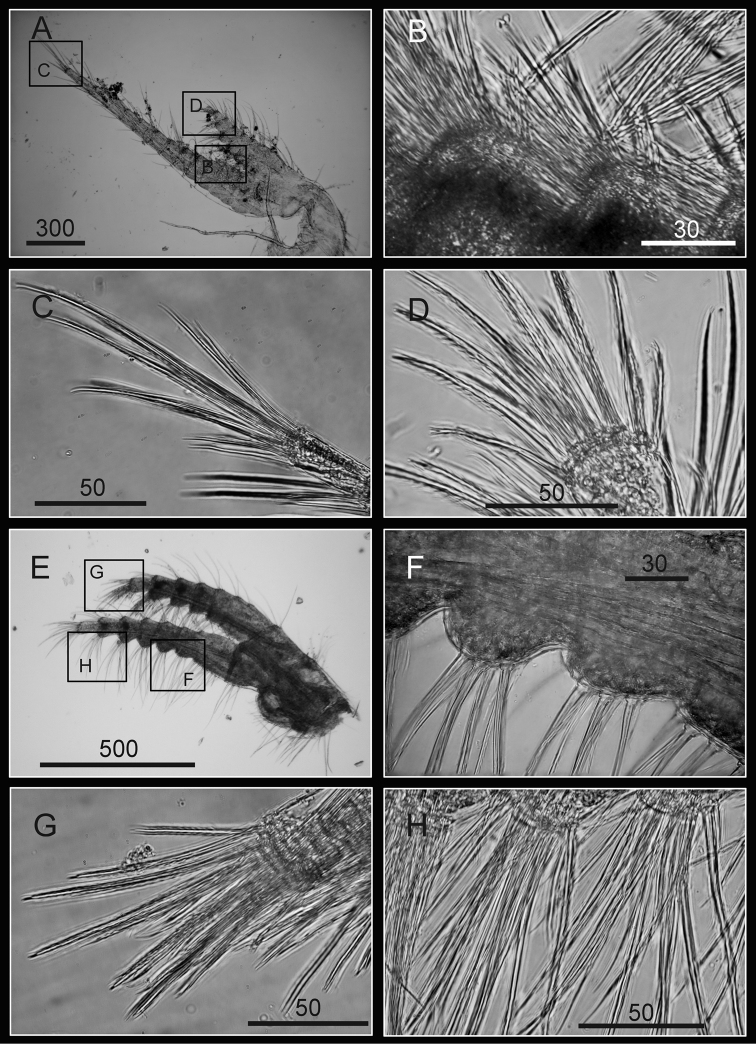
Cirri of *Darwiniella angularis* sp. n. **A** Cirrus I (NMNS-6878-001) **B** Serrulate setae on anterior ramus **C** Serrulate setae on anterior ramus apex **D** Serrulate setae on posterior ramus apex **E**  Cirrus II (CEL-TI-1-7) **F** Serrulate setae on posterior ramus **G** Serrulate setae on anterior ramus apex **H** Serrulate setae on posterior ramus base. (scale bar: μm)

**Figure 8. F8:**
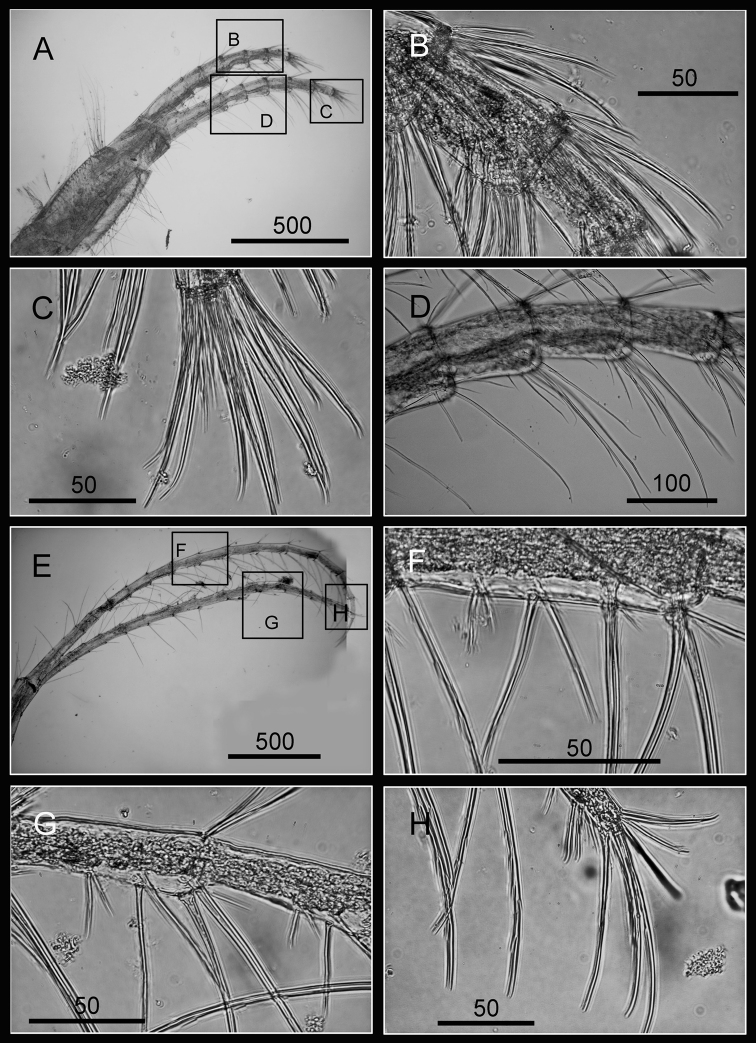
Cirri of *Darwiniella angularis* sp. n. **A** Cirrus III (NMNS-6878-001) **B** Serrulate setae on anterior ramus **C** Serrulate setae distally **D** Serrulate setae on posterior ramus **E** Cirrus IV (NMNS-6878-001) **F** Intermediate segment with 4 pairs of serrulate setae **G** Intermediate segment with serrulate setae **H** Serrulate setae distally. (scale bar: μm)

**Figure 9.**
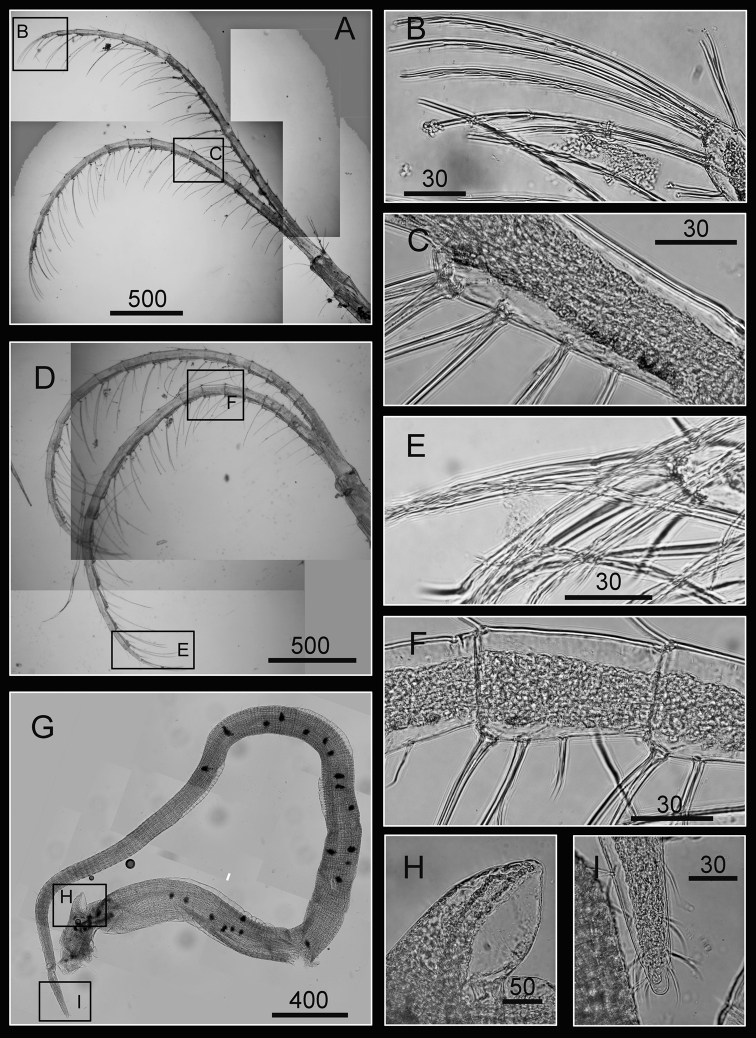
Cirri and penis of *Darwiniella angularis* sp. n. **A** Cirrus V (NMNS-6878-001) **B** Serrulate setae on apex **C** Intermediate segment with 4 pairs of serrulate setae **D** Cirrus VI (NMNS-6878-001) **E**  Serrulate setae distally **F** Intermediate segment with 4 pairs of serrulate setae **G** Penis (CEL-TI-9-10) **H** Basi-dorsal point of penis **I** Apex of penis. (scale bar: μm)

Cirrus I with rami unequal, anterior ramus long, slender, 14-segmented, posterior ramus 6-segmented (Figs 6A, 7A), bearing serrulate setae (Fig. 7B, C, D). Cirrus II with rami subequal, both 7-segmented (Figs 6B, 7E), bearing serrulate setae (Fig. 7F, G, H). Cirrus III with rami subequal, anterior ramus 8-segmented, posterior ramus 7-segmented (Figs 6C, 8A), bearing serrulate setae (Fig. 8B, C, D). Cirri IV–VI long, slender, rami equal. Cirrus IV with anterior ramus16-segmented, posterior 15-segmented, Cirrus V (anterior 21-segmented, posterior 19-segmented), Cirrus VI (anterior 22-segmented, posterior 21-segmented) (Figs 6D, E, F, 8E, 9A, D). Intermediate segments of Cirri IV–VI with 4 pairs of serrulate setae (Figs 8F, 9C, F), distal pair longest, proximal pair shortest.

Penis long (1.9 times length of Cirrus VI), annulated, some dark spots present, scattered short simple setae (Fig. 9G). Pedicel with basidorsal point (Fig. 9G, H), apex bearing short simple setae (Fig. 9I).

##### Distribution.

At present only recorded from Taiwan.

##### Etymology.

The name *angularis* denotes the presences of the obvious adductor plate angle, which is a diagnostic character of this species.

##### Remarks.

External shell morphology and arthropodal characters of *Darwiniella angularis* sp. n. are similar to *Darwiniella conjugatum*. However, *Darwiniella angularis* has an obvious adductor plate angle, whereas that of *Darwiniella conjugatum* is less obvious (see description below). *Darwiniella conjugatum* also has a sharper spur angle than *Darwiniella angularis* - mean ± 1 SD of the spur angle (from 10 specimens) reaching 23.6 ± 4.8^o^ in *Darwiniella conjugatum* and 32 ± 4.6^o^ in *Darwiniella angularis*, which is significantly different between the two species using the t-test (t value: -4.3, df = 18, p <0.05).

#### Darwiniella conjugatum

(Darwin, 1854)

http://species-id.net/wiki/Darwiniella_conjugatum

**Figure 10.**
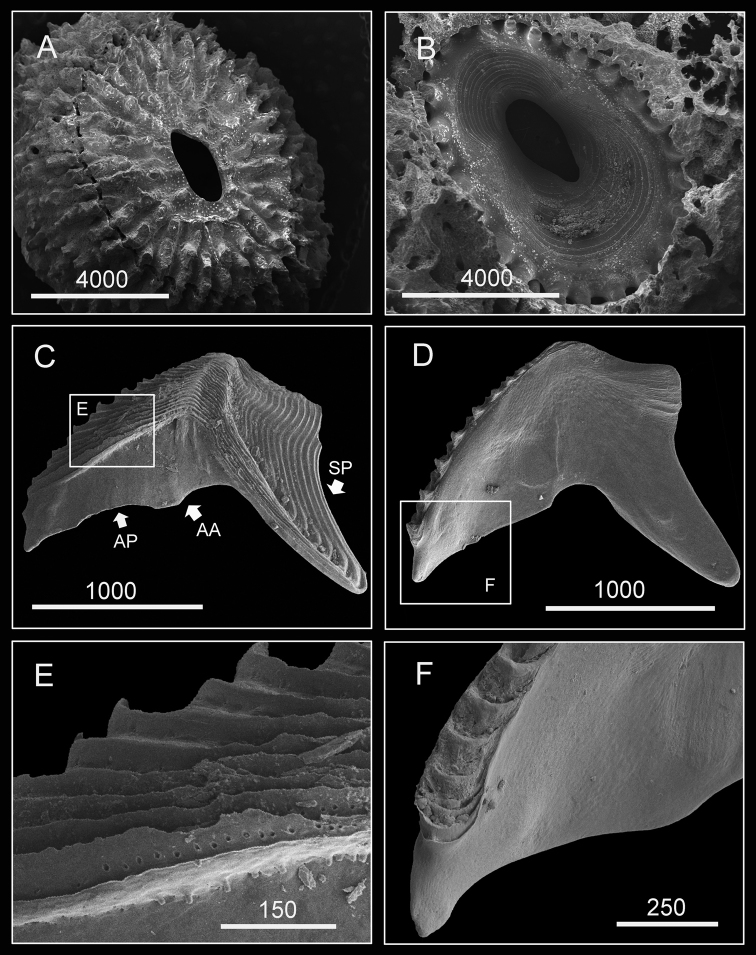
Hard parts of *Darwiniella conjugatum* (Darwin, 1854) (CEL-RYU-28-1, CEL-RYU-47-4, CEL-RYU-170-1) **A** Dorsal view of shell **B** Ventral view of shell **C** Dorsal view of fused scutum and tergum **D** Ventral view of fused scutum and tergum **E** Horizontal striations on dorsal surface of scutum **F** Occludent margin base and rostral tooth. (**AP**: adductor plate, **AA**: adductor plate angle, **SP**: spur, scale bar: μm)

Figs 10 [Fig F11] [Fig F12] [Fig F13] [Fig F14] [Fig F15] [Fig F16] –17

Pyrgoma conjugatum Darwin 1854: 364–365, pl. 12, fig. 7a–c.; Weltner 1897: 255; Gruvel 1905: 306–309; Annandale 1906: 143; Hoek 1913: 264; Broch 1922: 344; Hiro 1931: 154; Sakakura 1934: tab. II (not seen); Hiro 1935: 47, 59–60, 66, 69, fig. 8a–c; Hiro 1937: 468 (not seen); Nilsson-Cantell 1938: 13, 66, tab. III; Broch 1947: 7; Kolosváry 1947: 427; Utinomi 1949: 69 (not seen).
Pyrgoma (Nobia) conjugatum Baluk and Radwanski 1967: 487.
Nobia conjugatum Ross and Newman 1973: 155, fig. 12d–e; Newman and Ross 1976: 58; Foster 1982: 209, tab. 5, pl. 1G, fig. 7F; Soong and Chang 1983: 244–245, fig. 3; Galkin 1986: 1292; Ren 1986: 146, pl. IV, 15–18; Ogawa and Matsuzaki 1990: tab. I (not seen); Anderson 1992: 306–309, figs 20–21, 37F; Ogawa et al. 1998: 3, fig. 2; Jones et al. 2000: 276; Jones 2003: tabs 6, 8; Poltarukha and Dautova 2004: 111, fig. 66.
Nobia conjugata Ogawa and Matsuzaki 1992: app. tab.
Darwiniella conjugatum Anderson 1992: 329, figs 38F, 39; Simon-Blecher et al. 2007: tabs I–II.
Darwiniella conjugata Asami and Yamaguchi 1997: 13–14, figs 1–2.

##### Materials examined.

CEL-RYU-28-1, Dafu Fishing Harbor, Siaoliouciou Island, Taiwan (22°20'N, 120°22'E), August 2010, coll. B.K.K. Chan, on coral host *Cyphastrea serailia* (Forskål, 1775). CEL-RYU-28-2 data same as CEL-RYU-28-1. CEL-RYU-38-4, Shanfu Fishing Harbor, Siaoliouciou Island, Taiwan (22°21'N, 120°21'E), August 2010, coll. B.K.K. Chan, on coral host *Cyphastrea serailia*. CEL-RYU-47-4 data same as CEL-RYU-38-4. CEL-RYU-66-1, Lobster Cave, Siaoliouciou Island, Taiwan (22°20'N, 120°23'E), August 2010, coll. B.K.K. Chan, on host coral *Cyphastrea* sp.. CEL-RYU-170-1, Shanfu Fishing Harbor, Siaoliouciou Island, Taiwan (22°21'N, 120°21'E), August 2010, coll. B.K.K. Chan, on coral host *Cyphastrea japonica*.

##### Diagnosis.

Scutum subtriangular, rostral tooth and obvious adductor plate present. Adductor plate angle not obvious. Tergum subtriangular, lateral depressor muscle crests, medial furrow and spur present. Spur triangular, long, curved and sharp.

##### Description.

Shell (8 mm in maximum basal diameter, rostro-carinal diameter to 6 mm) plates fully fused, purple, oval, externally surface with about 25 strip-like projections differing in length and radiating from nearly subcentral orifice to plate margin (Fig. 10A). Bases of shell with about 27 internal ribs radiating from the rim of the inner operculum to the basal margin of the shells (Fig. 10B). Orifice oval, long and narrow, about 3/8 length of rostro-carinal diameter.

Scutum and tergum white, fused without any junctions (Fig. 10C, D). Scutum subtriangular, width 1.5 times length of height, occludent margin slightly curved, with a rostral tooth on bottom (Fig. 10F) and continuous teeth along the ventral surface of occludent margin, tooth size increasing gradually from apex to base. Ventral view with an apparent oval-shaped adductor muscle scar (Fig. 10D). Dorsal view with obvious adductor plate, extending below basal margin, plate more than 1/2 height of scutum. Basal margin of adductor plate without obvious adductor plate angle (Fig. 10C). Dorsal surface of scutum with horizontal striations, striations with row of small pores (Fig. 10E).

Tergum subtriangular, lateral depressor muscle crests present. Spur triangular, long, curved and sharp, height reaching more than 1/2 height of tergum, basal margin not obvious due to the curved spur. Dorsal surface with medial furrow, curving from basal margin towards the carinal margin of tergum, width of furrow increased gradually from apex to base (Fig. 10C). Dorsal surface with horizontal striations, striations with row of small pores.

**Figure 11. F11:**
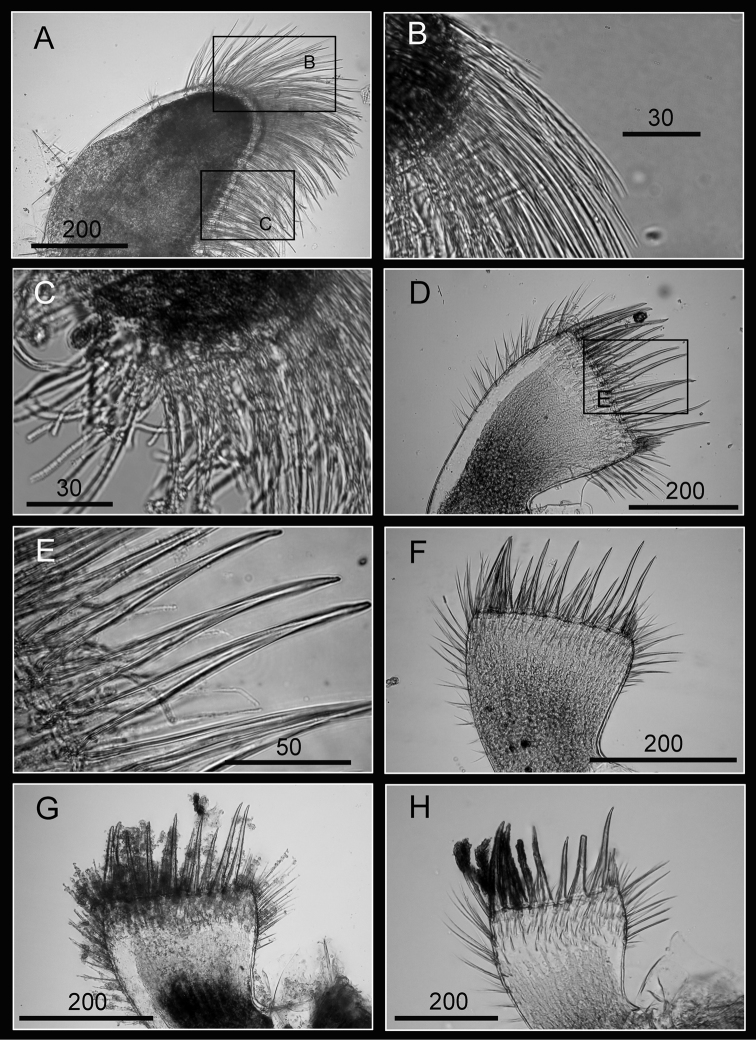
Oral cone of *Darwiniella conjugatum* (Darwin, 1854) **A** Maxilla (CEL-RYU-28-2) **B** Serrulate setae on apex **C** Serrulate setae on inferior margin **D** Maxillule (CEL-RYU-28-2) **E** Large simple type setae on cutting edge **F** Maxillule (additional specimen, CEL-RYU-28-1) **G** Maxillule (additional specimen, CEL-RYU-38-4) **H** Maxillule (additional specimen, CEL-RYU-66-1). (scale bar: μm)

**Figure 12. F12:**
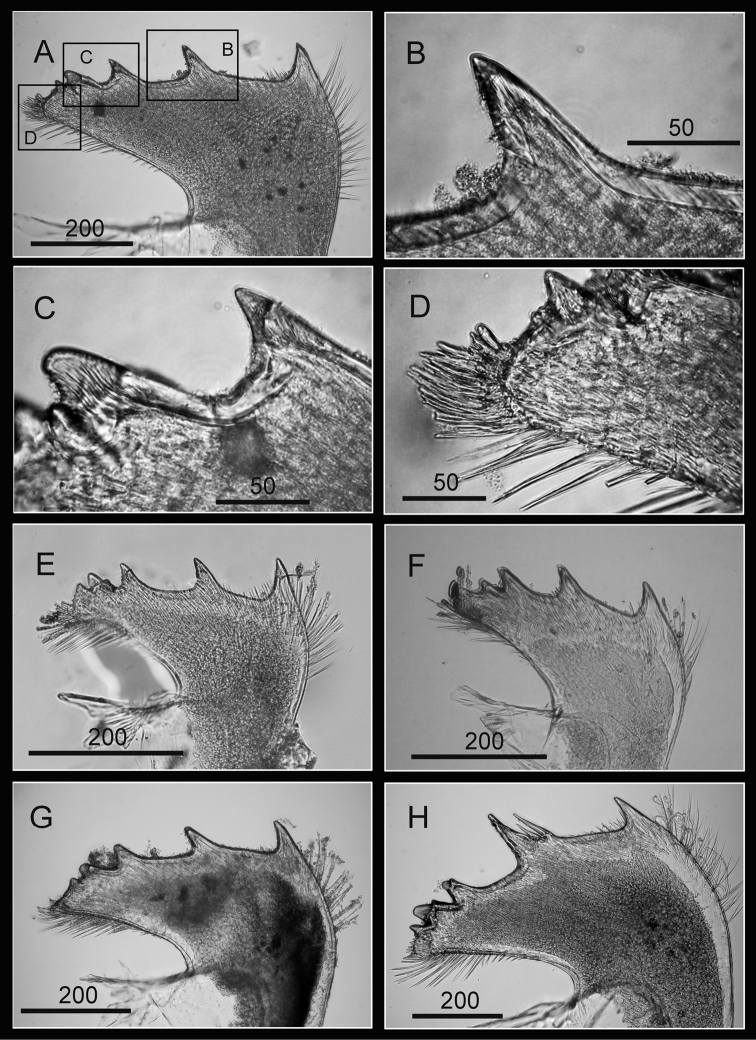
Oral cone of *Darwiniella conjugatum* (Darwin, 1854) **A** Mandible (CEL-RYU-28-1) **B** Bidentate second tooth **C** Bidentate third and fourth tooth **D** Inferior angle with simple type seta **E**  Mandible (additional specimen, CEL-RYU-170-1) **F** Mandible (additional specimen, CEL-RYU-66-1) **G** Mandible (additional specimen, CEL-RYU-38-4) **H** Mandible (additional specimen, CEL-RYU-28-2). (scale bar: μm)

**Figure 13. F13:**
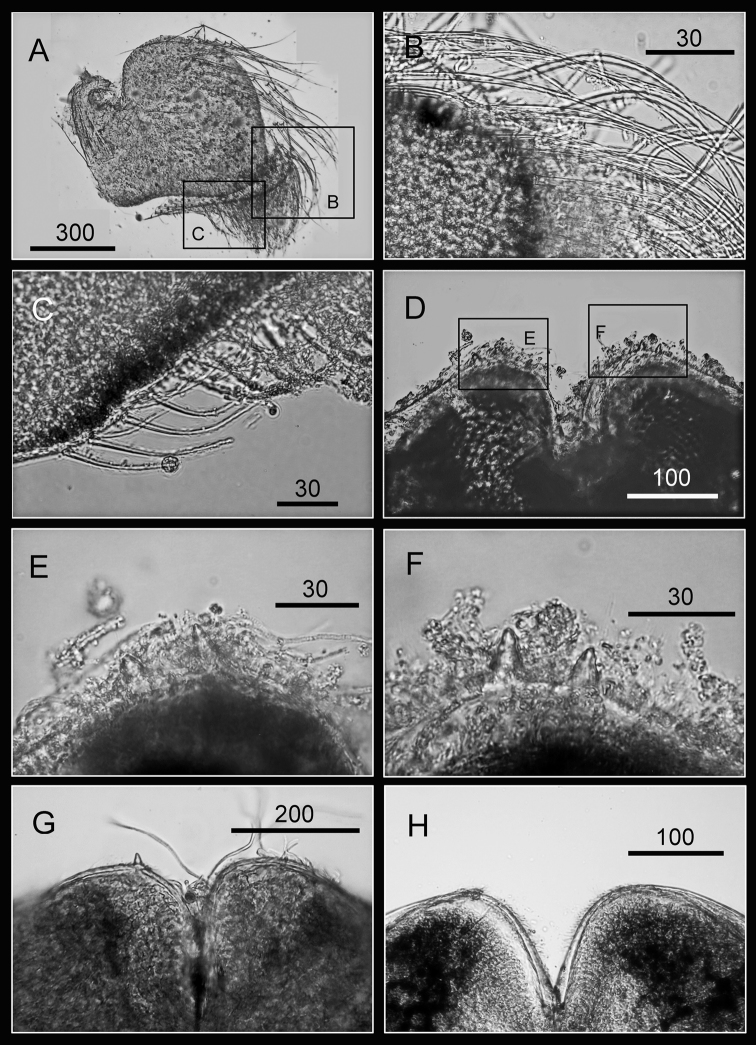
Oral cone of *Darwiniella conjugatum* (Darwin, 1854) **A** Mandibular palp (CEL-RYU-66-1) **B** Serrulate setae distally **C** Serrulate setae on inferior margin **D** Labrum (CEL-RYU-38-4) **E** Teeth on labrum **F** Teeth on labrum **G** Labrum (additional specimen, CEL-RYU-66-1) **H** Labrum (additional specimen, CEL-RYU-28-1). (scale bar: μm)

Maxilla oval (Fig. 11A), serrulate setae distally (Fig. 11B) and along inferior margin (Fig. 11C). Maxillule cutting edge straight, without notch, bearing row of 13 large, similar sized setae (consistent through 2 specimens, Fig. 11D, F, and inconsistent through 2 specimens with 11 and 9 large setae, Fig. 11G, H, respectively). Region close to cutting edge with dense fine simple type setae (Fig. 11E), anterior and posterior margin with long simple type setae. Mandible with five teeth (excluding inferior angle) (teeth number consistent in five specimens but differed in morphologies, Fig. 12A, E, F, G, H). Second, third, fourth and fifth teeth bidentated (Fig. 12B, C), the first 4 teeth occupying most of length of cutting edge. Lateral surface, lower margin and cutting edge of mandible bearing simple type setae. Lower margin short, about 1/20 length of total length of mandible, inferior angle ending in a blunt angle with dense, fine setae (Fig. 12D). Mandibular palp rectangular, elongated (Fig. 13A), bearing serrulate setae distally (Fig. 13B) and on inferior margin (Fig. 13C). Labrum bilobed, with V-shaped notch between 2 lobes, 2 sharp teeth on each side of notch (inconsistent in three specimens, Fig. 13D, G, H).

**Figure 14. F14:**
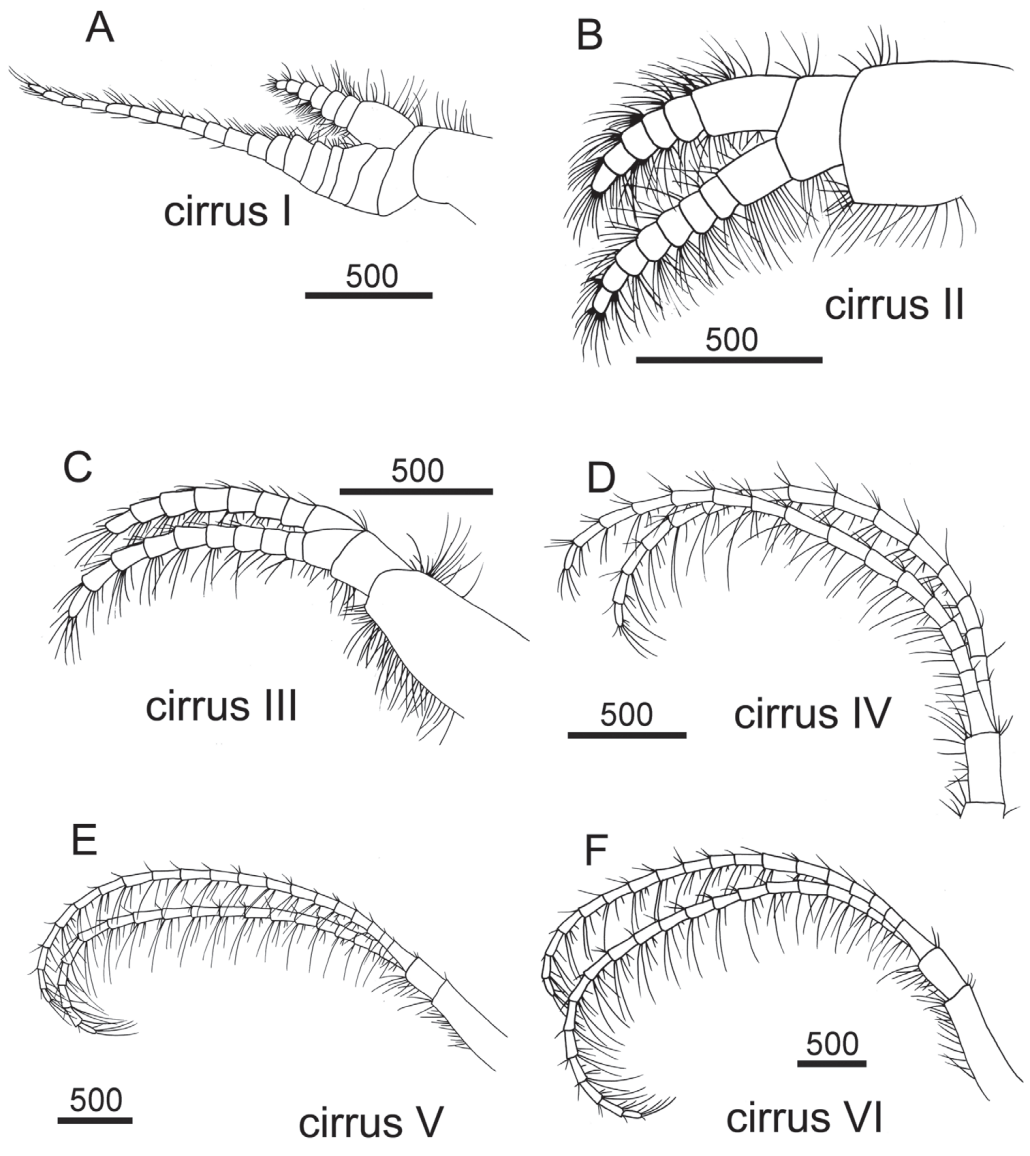
Line drawing of *Darwiniella conjugatum* (Darwin, 1854) **A** Cirrus I **B** Cirrus II **C** Cirrus III **D** Cirrus IV **E** Cirrus V **F** Cirrus VI. (scale bar: μm).

**Figure 15. F15:**
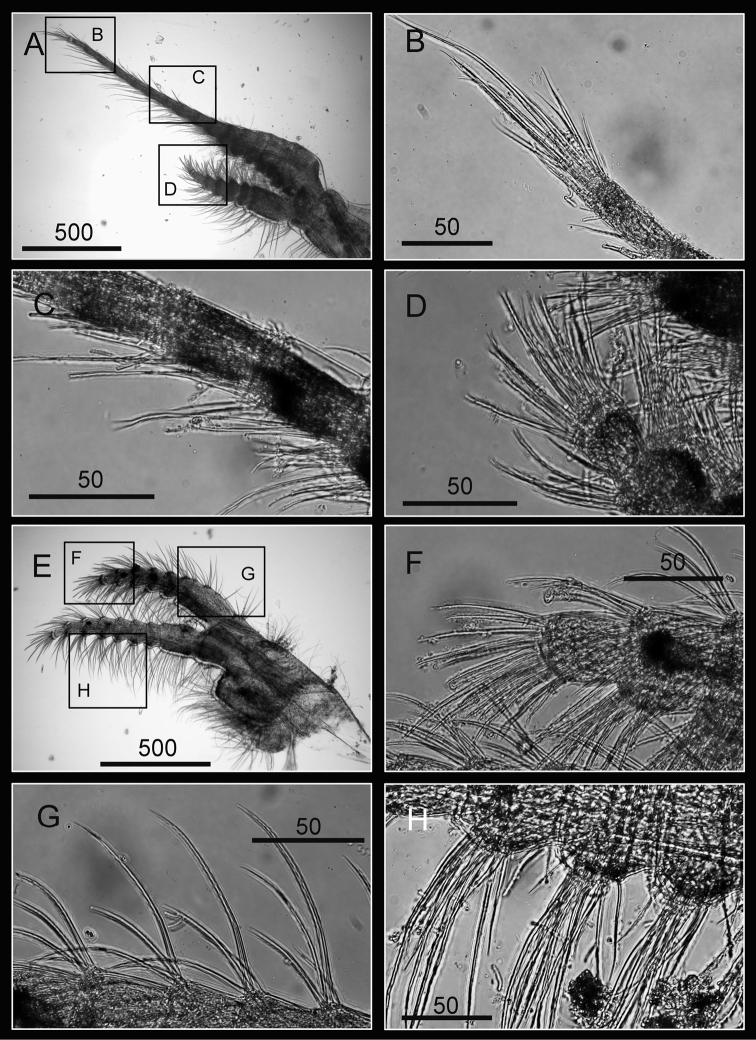
Cirri of *Darwiniella conjugatum* (Darwin, 1854) **A** Cirrus I (CEL-RYU-28-2) **B** Serrulate setae on anterior ramus apex **C** Serrulate setae on anterior ramus **D** Serrulate setae on posterior ramus apex **E** Cirrus II (CEL-RYU-28-2) **F** Serrulate setae on anterior ramus apex **G** Serrulate setae on anterior ramus **H** Serrulate setae on posterior ramus apex. (scale bar: μm)

**Figure 16. F16:**
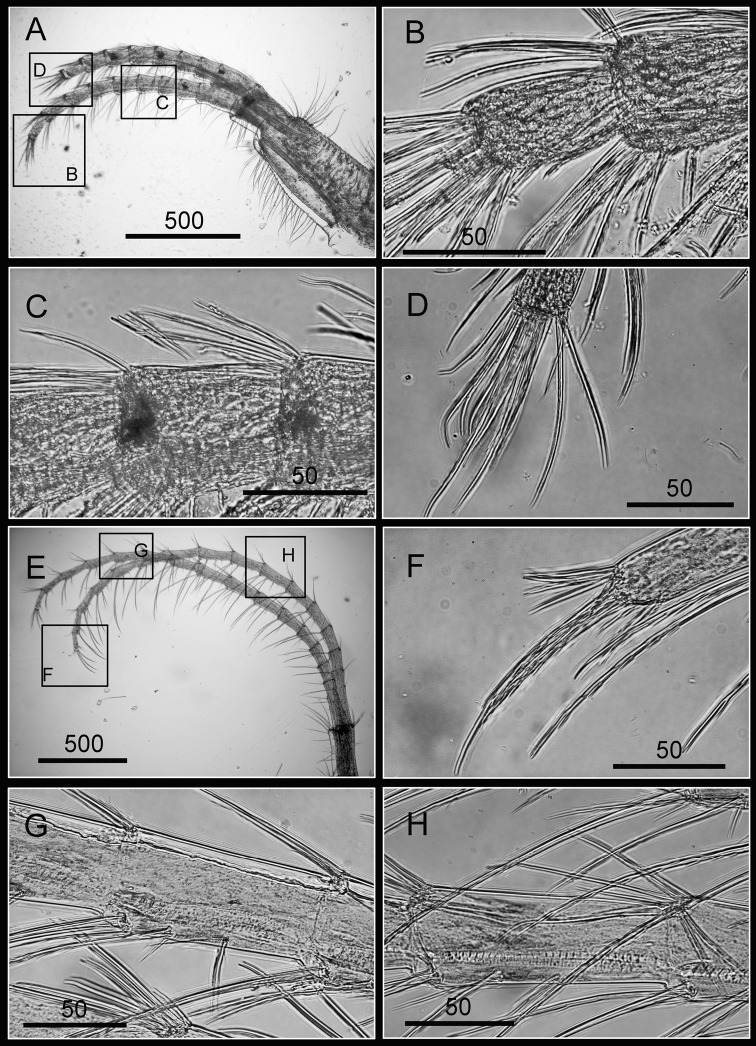
Cirri of *Darwiniella conjugatum* (Darwin, 1854) **A** Cirrus III (CEL-RYU-28-2) **B** Serrulate setae on posterior ramus **C** Serrulate setae on posterior ramus **D** Serrulate setae distally **E** Cirrus IV (CEL-RYU-28-2) **F** Serrulate setae on apex **G** Intermediate segment with 4 pairs of serrulate setae **H** Intermediate segment with serrulate setae. (scale bar: μm)

**Figure 17.**
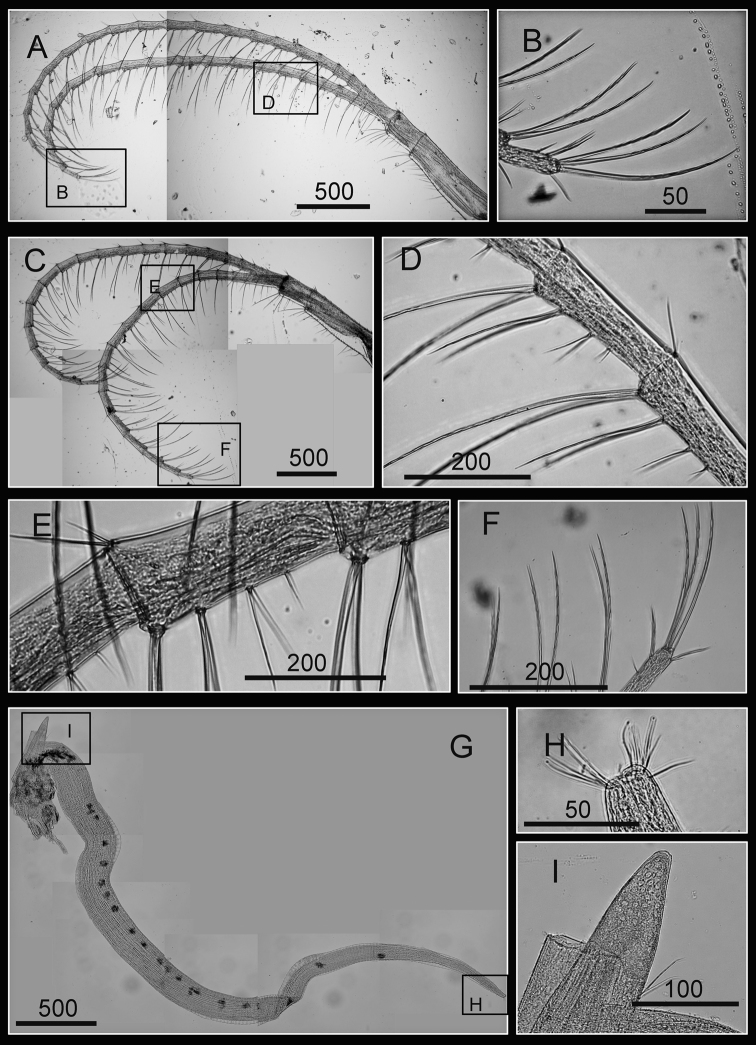
Cirri and penis of *Darwiniella conjugatum* (Darwin, 1854) **A** Cirrus V (CEL-RYU-28-2) **B** Serrulate setae on apex **C** Cirrus VI (CEL-RYU-28-2) **D** Intermediate segment with 4 pairs of serrulate setae **E** Intermediate segment with 4 pairs of serrulate setae **F** Serrulate setae on apex **G** Penis (CEL-RYU-170-1) **H** apex of penis **I** Basidorsal point of penis. (scale bar: μm)

Cirrus I with rami unequal, anterior ramus long and slender, with 17-segmented, posterior ramus 7-segmented (Figs 14A, 15A), bearing serrulate setae (Fig. 15B, C, D), dark spots exist on each segment of the ramus (Fig. 15A). Cirrus II with rami almost equal, anterior ramus 8-segmented and posterior ramus 6-segmented (Figs 14B, 15E), bearing serrulate setae (Fig. 15F, G, H), dark spots exist on each segment of the ramus (Fig. 15E). Cirrus III rami equal, anterior ramus 10-segmented, posterior ramus 8-segmented (Figs 14C, 16A), bearing serrulate setae (Fig. 16B, C, D), dark spots exist on each segment of the ramus (Fig. 16A). Cirrus IV–VI long, slender, with equal rami size. Number of segments on Cirrus IV (anterior 16, posterior 16), Cirrus V (21, 20), Cirrus VI (21, 18) (Figs 14D, E, F, 16E, 17A, C). Intermediate segments of Cirrus IV–VI with four pairs of serrulate setae (Figs 16G, 17D, E), distal pair longest, proximal pair shortest. Dark spots exist on each segment of the ramus (Figs 16E, 17A, C).

Penis long (1.2 times length of Cirrus VI), annulated, some dark spots present, scattered short simple type setae (Fig. 17G). Pedicel with basidorsal point (Fig. 17G, I), apex bearing short simple type setae (Fig. 17H).

##### Distribution.

South China Sea: Singapore, Mainland China (Hong Kong), Vietnam (Nhatrang Bay).Pacific Ocean: Taiwan (Green Island, Turtle Island, Siaoliouciou Island, Kenting, Suao), Japan, Australia (Western Australia & Great Barrier Reef),Philippine.Indian Ocean: Mauritius (Albion), Bay of Bengal, Thailand (Gulf of Siam), Sri Lanka,Red Sea.

##### Remarks.

*Darwiniella conjugatum* is widely reported in the Indo-Pacific Ocean. Note Ogawa and Matsuzaki (1992) and Asami and Yamaguchi (1997) misspelled the species name *Darwiniella conjugatum* as ‘*conjugata*’.

### Molecular analysis

**Figure 18.**
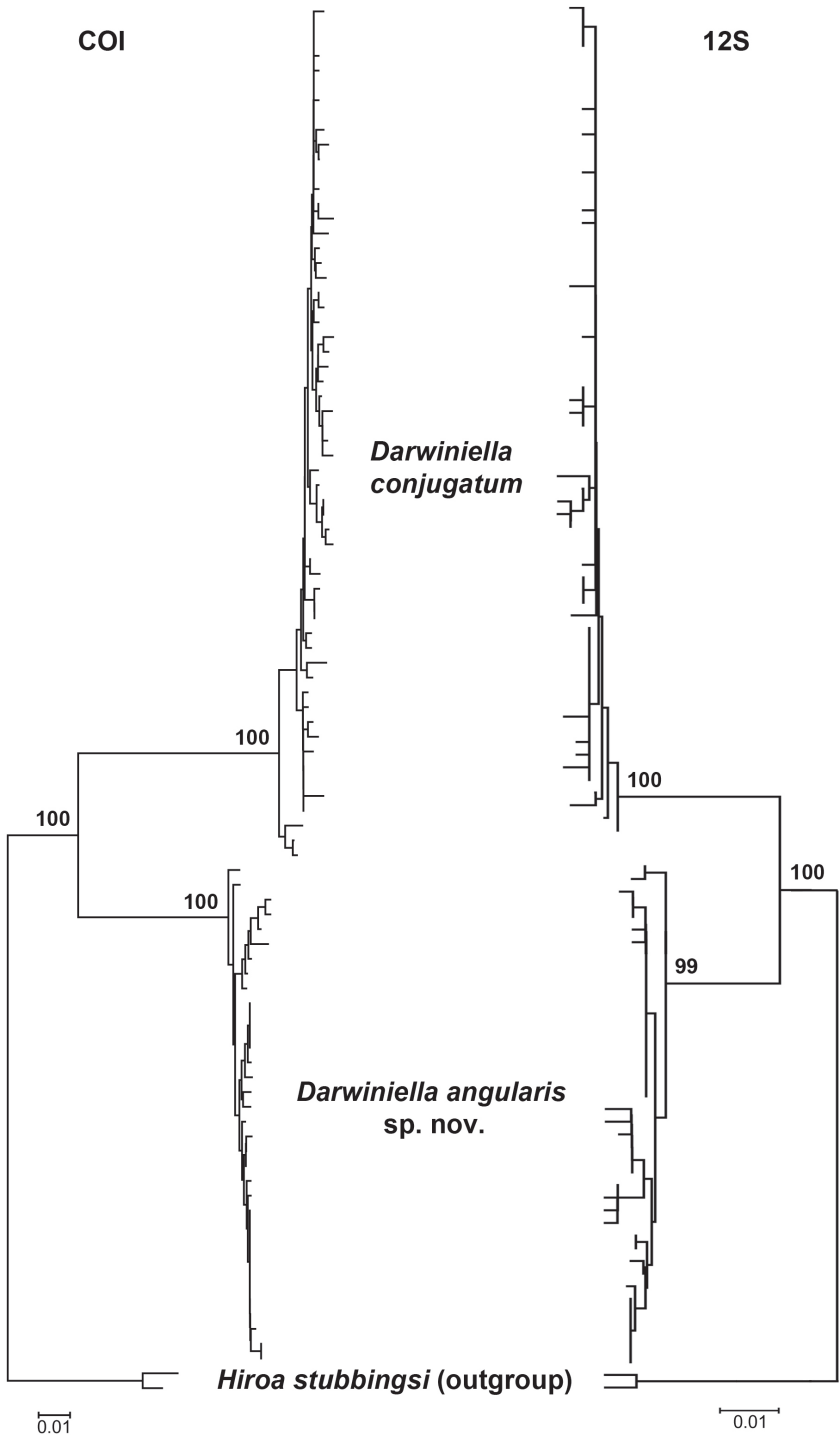
Neighbor-Joining method inferred genealogical relationships of *Darwiniella* specimens based on 472bp 12S and 642bp COI with *Hiroa stubbingsi* as the outgroup. Numbers above the major nodes are bootstrap values of 1000 replicates.

After trimming and aligning the sequences, 472bp of 12S rDNA and 642bp of COI were obtained from 107 and 92 *Darwiniella* specimens without indels, respectively (see on-line Appendix 2: Table 2 for GenBank accession numbers, on-line Appendix 3: alignment Figure). The NJ inferred genealogical relationships based on 12S and COI were congruent to each other (Fig. 18). Both datasets showed the presence of two well-supported distinct lineages, corresponding to *Darwiniella conjugatum* and *Darwiniella angularis* sp. n.. In 12S, 37 out of the 69 variable nucleotide sites were parsimony informative. Evolutionary distances based on p-distance/K2P-distance were 0.004/0.004 and 0.006/0.006 within *Darwiniella conjugatum* and *Darwiniella angularis* sp. n., respectively, and 0.056/0.058 between the two species. In COI, 100 out of the 127 variable nucleotide sites were parsimony informative. Evolutionary distances based on p-distance/K2P-distance were 0.010/0.010 and 0.006/0.006 within *Darwiniella conjugatum* and *Darwiniella angularis* sp. n., respectively, and 0.120/0.132 between species.

## Discussion

In the present study, a new species is identified from the previously monotypic genus *Darwiniella* that exhibit diagnostic morphological and molecular differences from *Darwiniella conjugatum*. These two species are difficult to distinguish from external shell morphology. The diagnostic characters are of the adductor plate angle of the scutum and the spur angle of the tergum.

Based on molecular analyses, the sequence distance of mitochondrial DNA markers 12S and COI within the two *Darwiniella* species (12S: 0. 4–0.6%, COI: 0.6–1.0%) is much smaller than between species (12S: 5.6–5.8%, COI: 12.0–13.2%). The sequence divergence of these two species has reached the level of congeneric species in another coral barnacle genus, *Cantellius* Ross & Newman, 1973 (12S: 5–7%, COI: 10–11 %) (Achituv et al. 2009), therefore further supporting *Darwiniella angularis* sp. n. as a separate species from *Darwiniella conjugatum*. Although the application of mitochondrial DNA in delimitating species can be limited by its natural history, e.g. reduced effective population size and introgression, and maternal inheritance (Rubinoff et al. 2006), the species boundary is evident based on the differentiation of 12S, COI and morphology of abundant samples from various localities.

Both *Darwiniella* species show significant host preference for the massive-form coral *Cyphastrea*. In the present study, 21 of 23 coral pieces with *Darwiniella conjugatum* embedded are *Cyphastrea* and only one is *Astreopora* (Appendix 2: Table 2). A similar pattern is observed in *Darwiniella angularis* where six of eight coral pieces are *Cyphastrea*, and only two are *Astreopora*. In previous studies, *Darwiniella conjugatum* has been recorded in *Cyphastrea* corals by Soong and Chang 1983 (Taiwan), Ren 1986 (Mainland China), Ogawa et al. 1998 (Mauritius) and Jones et al. 2000 (South China Sea), agreeing with the current study. However, Ogawa and Matsuzaki (1992) also recorded *Darwiniella conjugatum* from *Cyphastrea*, *Goniopora* de Blainville, 1830 and *Favites* Link, 1807 species in Japan.

## Supplementary Material

XML Treatment for Darwiniella angularis

XML Treatment for Darwiniella conjugatum

## Appendix 1

**Table 1:** Site and sampling location details. (doi: 10.3897/zookeys.214.3291.app1) File format: MS Word document(docx).

**Explanation note:** Study sites and sampling locations for *Darwiniella* in the present study.

## Appendix 2

**Table 2:** GenBank accession numbers for the barnacle species used in the present study. (doi: 10.3897/zookeys.214.3291.app2) File format: MS Excel spreadsheet (xls).

**Explanation note:** GenBank accession numbers for the barnacle species used in the present study.

## Appendix 3

**Figure 1:** Sequence alignment of (a) 472 bp 12S rDNA and (b) 642bp COI. Matching characters to the first sequence are labeled as dots. (doi: 10.3897/zookeys.214.3291.app3) File format: MS Word document(docx).

**Explanation note:** Sequence alignment of (a) 472 bp 12S rDNA and (b) 642bp COI. Matching characters to the first sequence are labeled as dots.
